# ANGPTL4 regulates ovarian cancer progression by activating the ERK1/2 pathway

**DOI:** 10.1186/s12935-024-03246-z

**Published:** 2024-02-04

**Authors:** Jiaqi Xu, Fei Wu, Yue Zhu, Tiantian Wu, Tianyue Cao, Wenxin Gao, Meng Liu, Weifeng Qian, Guannan Feng, Xiaoxue Xi, Shunyu Hou

**Affiliations:** 1 Department of Obstetrics and Gynecology, The Affiliated Suzhou Hospital of Nanjing Medical University, Gusu School, Nanjing Medical University; Suzhou Municipal Hospital, No.26, Daoqian Street, Suzhou, 215002 Jiangsu China; 2 Department of Breast and Thyroid Surgery, The Affiliated Suzhou Hospital of Nanjing Medical University, Gusu School, Nanjing Medical University; Suzhou Municipal Hospital, No.26, Daoqian Street, Suzhou, 215002 Jiangsu China; 3 State Key Laboratory of Reproductive Medicine and Offspring Health, Department of Histology and Embryology, Nanjing Medical University, Nanjing, China

**Keywords:** Ovarian cancer, ANGPTL4, ERK1/2, Tumorigenesis

## Abstract

**Background:**

Ovarian cancer (OC) has the highest mortality rate among all gynecological malignancies. A hypoxic microenvironment is a common feature of solid tumors, including ovarian cancer, and an important driving factor of tumor cell survival and chemo- and radiotherapy resistance. Previous research identified the hypoxia-associated gene angiopoietin-like 4 (ANGPTL4) as both a pro-angiogenic and pro-metastatic factor in tumors. Hence, this work aimed to further elucidate the contribution of ANGPTL4 to OC progression.

**Methods:**

The expression of hypoxia-associated ANGPTL4 in human ovarian cancer was examined by bioinformatics analysis of TCGA and GEO datasets. The CIBERSORT tool was used to analyze the distribution of tumor-infiltrating immune cells in ovarian cancer cases in TCGA. The effect of ANGPTL4 silencing and overexpression on the proliferation and migration of OVCAR3 and A2780 OC cells was studied in vitro, using CCK-8, colony formation, and Transwell assays, and in vivo, through subcutaneous tumorigenesis assays in nude mice. GO enrichment analysis and WGCNA were performed to explore biological processes and genetic networks associated with ANGPTL4. The results obtained were corroborated in OC cells in vitro by western blotting.

**Results:**

Screening of hypoxia-associated genes in OC-related TCGA and GEO datasets revealed a significant negative association between ANGPTL4 expression and patient survival. Based on CIBERSORT analysis, differential representation of 14 distinct tumor-infiltrating immune cell types was detected between low- and high-risk patient groups. Silencing of ANGPTL4 inhibited OVCAR3 and A2780 cell proliferation and migration in vitro and reduced the growth rate of xenografted OVCAR3 cells in vivo. Based on results from WGCNA and previous studies, western blot assays in cultured OC cells demonstrated that ANGPTL4 activates the Extracellular signal-related kinases 1 and 2 (ERK1/2) pathway and this results in upregulation of c-Myc, Cyclin D1, and MMP2 expression. Suggesting that the above mechanism mediates the pro-oncogenic actions of ANGPTL4T in OC, the pro-survival effects of ANGPTL4 were largely abolished upon inhibition of ERK1/2 signaling with PD98059.

**Conclusions:**

Our work suggests that the hypoxia-associated gene ANGPTL4 stimulates OC progression through activation of the ERK1/2 pathway. These findings may offer a new prospect for targeted therapies for the treatment of OC.

**Supplementary Information:**

The online version contains supplementary material available at 10.1186/s12935-024-03246-z.

## Introduction

Due to its highly aggressive nature, drug resistance, and susceptibility to distant metastases, ovarian cancer (OC) has the highest mortality rate among all gynecological malignancies [1, 2]. A hypoxic microenvironment is a common feature of tumors, including OC [3, 4]. Median oxygen levels in hypoxic intratumoral environments are < 2%, i.e. much lower than those present in normal tissues (~ 5%) [5]. Hypoxia is an important driver of tumorigenesis, as it promotes angiogenesis, induces stem-like phenotypes in tumor cells, and allows them to acquire greater proliferative and invasive capabilities [6, 7]. Under hypoxic conditions, activation of hypoxia-associated genes acting on various signal transduction cascades serves to regulate cell proliferation, migration, and apoptosis in tumor cells. Therefore, the study of genes associated with hypoxic states are of great value to design more efficient anticancer therapies [8, 9].

Previous studies have suggested that ANGPTL4 is associated with hypoxia [10]. ANGPTL4 is a secreted glycoprotein, 45 ~ 65 kDa in size, with an N-terminal convoluted structural domain (N-ANGPTL4) and a C-terminal fibrinogen-like domain (C-ANGPTL4) linked by a linker region [11, 12]. Some studies have shown that the N-ANGPTL4 domain participates in the regulation of lipid metabolism, while the C-ANGPTLL4 domain may be a regulator of the tumorigenic process [11]. Previous studies have suggested that ANGPTL4 has great potential as a molecular marker in tumor diagnosis and patient prognosis, as it regulates tumor progression through various signaling pathways [13–15], including Extracellular signal-related kinases 1 and 2 (ERK1/2) [16, 17]. In OC, it has been shown that ANGPTL4 reduces angiogenesis, thereby inhibiting tumor growth [18]. Although studies on the mechanisms by which ANGPTL4 regulates OC development and progression are still scarce, the above evidence suggests that ANGPTL4 may serve as a molecular marker and therapeutic target for OC diagnosis and treatment.

The aim of this investigation was to explore the involvement of ANGPTL4, which is associated with hypoxia, in the proliferation and migration of ovarian cancer (OC) cells. Initially, we employed bioinformatics analysis to anticipate the potential participation of the ERK1/2 pathway in the regulation of ANGPTL4 in OC cell lines (OVCAR3 and A2780), with a specific focus on downstream targets such as c-Myc, CyclinD1, and MMP2. Importantly, no previous research has examined the correlation between ANGPTL4 and the ERK1/2 pathway. Consequently, our findings furnish fresh evidence that the ANGPTL4-ERK1/2 signaling axis influences the progression of OC by targeting c-Myc, CyclinD1, and MMP2. These novel insights into the regulatory mechanisms underlying ANGPTL4 in OC progression have the potential to contribute to the development of innovative approaches for the treatment of OC.

## Materials and methods

### Screening of hypoxia-related genes

A total of 200 genes related to hypoxia were obtained through the Gene Set Enrichment Analysis (GSEA) website (https://www.gsea-msigdb.org/gsea/index.jsp). Clinical prognostic information from OC patient samples in the Cancer Genome Atlas (TCGA) (https://portal.gdc.cancer.gov/) and corresponding expression values for the above 200 genes were downloaded and collated. With overall survival time (OS) as the outcome variable, univariate COX regression analysis was used to screen the 200 hypoxia-related genes in 53 TCGA-OC clinical samples. Significant OS-predictive genes (p-value < 0.05) were presented by drawing a forest plot. In GSE18520 (https://www.ncbi.nlm.nih.gov/gds/?term=GSE18520), Kaplan–Meier survival plots were drawn for the genes screened after univariate COX regression analysis through the Kaplan–Meier plotter website. Genes with adverse effects on the prognosis of OC patients were selected for subsequent analysis.

### Clinical samples

Seventeen matched cancerous and paracancerous tissue specimens were collected from OC patients with surgical resection from January 2021 to January 2022 in our hospital. None of patients received specific treatment before undergoing surgical resection. Informed consent was obtained from the patients before surgery. The study was approved by the Ethical Committee of The Affiliated Suzhou Hospital of Nanjing Medical University (KL901365). Collected tissues were stored in RNA Keeper Tissue Stabilizer (Vazyme, Nanjing, China) at − 80 °C. The tissues were pulverized into a fine powder using liquid nitrogen after being treated with RNA Keeper Tissue Stabilizer.

### Single-cell RNA-seq data analysis and CIBERSORT screening

The GSE184880 dataset (https://www.ncbi.nlm.nih.gov/gds/?term=GSE184880), containing single-cell RNA sequencing (scRNA-seq) information, was downloaded from the GEO database. The ‘Seurat’ R package was used to analyze scRNA-seq data from two normal ovarian tissue samples and four OC tissue samples selected from GSE184880. From this analysis, cells with ≤ 20% of mitochondrial genes were retained. Simultaneously, the cells with number of genes (nFeature RNA) ≤ 300 or ≥ 5000 were filtered out. The data was normalized by the ‘LogNormalize’ method after scaling the gene expression level, and then the ‘vst’ method was utilized to identify 3000 highly variable genes (HVGs) within each sample. We used the SCTransform function for pre-processing and to reduce the batch effect. By combining with the elbow plot and selecting the inflection point and the principal component (PC) with a smooth curve, we selected the first 20 dimensions for follow-up analysis. The’SingleR’ package was used for annotation of cell clusters.

CIBERSORT provides an estimation of the abundance of immune cell types present in the tumor environment. OC samples from TCGA were separated into two groups based on the median expression of ANGPTL4, and the infiltration of immune cells in each group was compared.

### Cell culture and transfection

Human OC cell lines (OVCAR3 and A2780) were purchased from Zhong Qiao Xin Zhou Biotechnology Company (Shanghai, China). The cell lines have been authenticated using Short Tandem Repeat (STR) analysis and performed regular mycoplasma-free tests. Both cell lines were cultured in RPMI-1640 medium (GIBCO, USA) containing fetal bovine serum (Excell Bio, New Zealand) and 1% penicillin/streptomycin (NCM Biotech, China) at 37 °C in a 5% CO_2_ atmosphere. A2780 cell lines required 10% FBS, while OVCAR3 cell lines required 20% FBS.

When cells were 60–70% confluent, we used Lipofectamine 2000 (Invitrogen, USA) to transfect negative control siRNA (si-NC) and ANGPTL4-targeted siRNA (si-ANGPTL4) (Tsingke Biotech Co., China) into cells. The siRNA sequences were as follows:

si-NC 5′-UUCUCCGAACGUGUCACGU -3′,

si-ANGPTL4 #1 5′-GCGAAUUCAGCAUCUGC-3′,

si-ANGPTL4 #2 5′-CAUGGAGGCUGGACAGU -3′.

An ANGPTL4 overexpression plasmid was constructed by Sangon Biotechnology Inc. (Shanghai, China). OC cells were transfected with X-treme GENE HP DNA Infection Reagent (Mannheim, Germany) using a plasmid-to-reagent ratio of 1:3. A subset of ANGPTL4-overexpressing cells was maintained in the presence of 20 µmol/L PD98059 (Selleck Chemicals) for 72 h, a selective inhibitor of the ERK1/2 signaling pathway.

### RNA extraction and RT-qPCR

Total RNA was extracted from cells with Trizol reagent and reversely transcribed into cDNA using HiScript III RT SuperMix for qPCR kit (Vazyme). The relative mRNA expression was measured using an AceQ qPCR SYBR Green Master Mix kit (Vazyme) on an Applied Biosystems 7500 RealTime PCR System. 18 s RNA (F: 5′- AAACGGCTACCACATCCAAG-3′; R: 5′- CCTCCAATGGATCCTCGTTA-3′) was used to normalize the relative expression of ANGPTL4 (F: 5′- ACTCAAGGCTCAGAACAGCA-3′; R: 5′- CAGCCTCTTTCTTCGGGCA -3′) based on the 2^−ΔΔ CT^ method.

### Western blotting

Cells were lysed with radioimmunoprecipitation assay (RIPA, Beyotime) buffer containing 1% phenylmethylsulfonyl fluoride (PMSF, Beyotime) as a protease inhibitor. Protein levels were quantified using a bicinchoninic acid (Beyotime Biotechnology) kit. The collected proteins were denatured through heating at 100 °C. From each sample, 15 μg of protein were separated by 10% sodium dodecyl sulfate–polyacrylamide gel electrophoresis (SDS-PAGE, GenScript, China), transferred to polyvinylidene difluoride membranes (PVDF, Millipore, Billerica, USA), blocked for 1 h in skimmed milk, and incubated with primary antibodies (Additional file 1: Table S1) overnight at 4 ºC. The next day, the membranes were washed with Tris-buffered saline with 0.1% Tween-20 (TBST) and treated with suitable secondary antibodies. Protein bands were visualized by an enhanced chemiluminescent substrate and quantified by Image-Pro Plus (Media Cybernetics, USA), as previously described [19, 20].

### Cell proliferation assay

Following transfection procedures, cells were seeded in 96-well plates (2500 cells per well). Cell viability was assessed with a Cell Counting Kit-8 assay (CCK8; Beyotime Biotechnology) and absorbance measurements performed at 450 nm on a microplate reader at 24 h intervals according to a routine procedure as previously described [21–23].

For colony-formation assays, 1000 cells were inoculated into individual wells of 6-well plates and cultured in complete medium, with weekly media changes, for 2 weeks. After 14 days, the cells were washed twice using phosphate-buffered saline (PBS) solution, fixed in methanol, and stained with a 0.1% crystal violet solution (Beyotime Biotechnology), as previously described [24, 25]. Finally, cell colonies were photographed and counted.

### Cell migration assay

Cell migration capacity was detected in Transwell chambers with 8 μm pore size membranes (Mil-lipore, Billerica, MA, USA) placed in a 24-well plate. A total of 45,000 cells in 300 μl of serum-free medium were seeded in the upper chamber, and 700 μl of complete medium was added to the lower chamber. After 48 h, the cells migrating to the membrane’s lower surface were fixed, stained and rinsed. Cells in random fields of view were imaged and counted.

### Mouse xenograft model

Seven-week-old female athymic BALB/c nude mice were maintained under specific pathogen-free conditions. OVCAR3 cells transfected with sh-NC and sh-ANGPTL4 were collected with a cell scraper. OVCAR3 xenografts were established by subcutaneously injecting 1 × 10^7^ cells resuspended in 100 µl of PBS into one side of the axilla. The right side of each mouse was injected with sh-NC-transfected cells, while the left side received sh-ANGPTL4-transfected cells. Tumor size (V = 0.5 × D × d^2^ (V, volume; D, longitudinal diameter; d, latitudinal diameter)) was measured every 3 days, and the mice were euthanized by cervical dislocation after 12 days. The tumors were then removed, weighed, measured, and photographed. This work was approved by the Animal Ethical and Welfare Committee of Nanjing Medical University.

### Immunofluorescence

Xenografted tumors were fixed in 4% paraformaldehyde for 48 h. The tumor tissues dehydrated with graded ethanol solutions, hyalinized with xylene, and then embedded in paraffin. Tissue sections were cut into 5 μm sections deparaffinized with xylene and rehydrated in a graded series of ethanol, as previously described [26–28]. Following antigen retrieval in sodium citrate buffer and blocking in 5% bovine serum albumin (BSA, w/v; Sunshine, Nanjing, China), sections were incubated with primary antibody against Ki67 (Abcam, Additional file 1: Table S1) overnight at 4 °C. The sections were subsequently washed and incubated with fluorescent secondary antibodies (Thermo Scientific, Waltham, MA, USA). Cell nuclei were stained with 4’,6-diamidino-2-phenylindole (DAPI, Beyotime Institute of Biotechnology), mounted, and imaged on a Zeiss laser fluorescence microscope (Zeiss LSM710, Carl Zeiss, Oberkochen, Germany).

### WGCNA and GO enrichment analysis

ANGPTL4-related differentially expressed genes (DEGs) in metastatic OC samples from GSE133296 dataset (https://www.ncbi.nlm.nih.gov/gds/?term=GSE133296) were analyzed using the WGCNA package in R programming language. When the soft-thresholding power was equivalent to 12, the correlation coefficient approached 0.85, and scale-free coexpression networks were constructed. The size of each gene module was limited to ≤ 30 genes, and cluster analysis was utilized to group genes based on similarities in their patterning. A cut-height of 0.4 was then applied to integrate similar modules. Correlations between phenotypic traits and module eigengenes were explored for identification of modules that were most closely linked to the expression of ANGPTL4.

Sample data transferred from GSE133296 were divided into high- and low-ANGPTL4 expression subgroups using the median of ANGPTL4 expression values as standard, and analyzed by the “limma” package in R. A screening-criteria set as |logFC|> 1, p < 0.05 yielded 491 DEGs. These DEGs were cross-analyzed with the genes in the most significant module in WGCNA, and GO enrichment analysis was performed on the overlapping genes.

### Statistical analysis

Statistical analyses were carried out using GraphPad Prism software. With at least three independent experiments, all values were presented as mean ± standard deviation (SD). Student’s t-test was used for analyzing differences between two groups, while one-way ANOVA was applied for multiple groups. P < 0.05 was considered significant.

## Results

### ANGPTLL4 expression is upregulated in OC

**Fig. 1:**
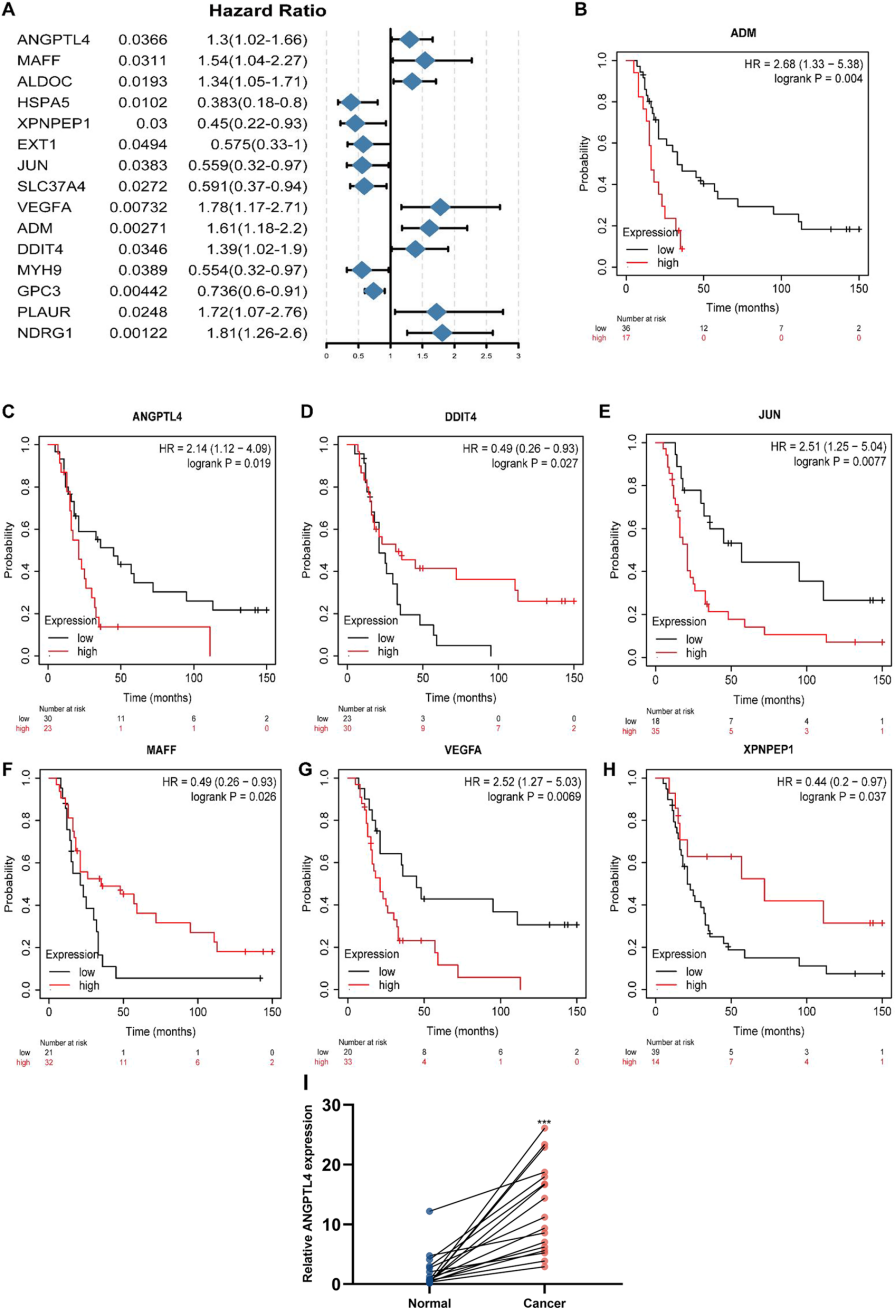
Screening for hypoxia-related genes associated with prognosis in OC. **A** Forest plots depicting results of survival analysis with univariate COX regression for 200 hypoxia-related genes in OC cases in TCGA. **B**–**H** Kaplan–Meier curves for OC patients in GSE18520. **I** Detection of ANGPTL4 expression by RT-qPCR in 17 paired cancerous and paracancerous tissues from OC patients. *p < 0.05, **p < 0.01, ***p < 0.001, compared with normal tissues

Univariate COX regression analysis of hypoxia-related genes in TCGA-OC specimens revealed 15 significant genes in association with patient survival. Among these, eight had an HR > 1, implying that they were hazardous to the outcome of OC patients (Fig. 1A). Kaplan–Meier survival curve analyses were further conducted on the screened genes in the GSE18520 dataset, and four genes with HR > 1 were detected (Fig. 1B–H). The combined results showed that the hypoxia-associated genes ANGPTL4, ADM, and VEGFA had a negative impact on the prognosis of OC. A previous study revealed that ADM upregulates HIF-1α and VEGF to promote angiogenesis in epithelial ovarian tumors [29]. Since the exact function of ANGPTL4 in ovarian carcinogenesis remains unclear, we conducted expression and functional analyses to investigate its potential role in OC. RT-qPCR was first used to examine ANGPTL4 transcription in 17 matched OC and tumor-adjacent tissue specimens from patients in our hospital. Results indicated that ANGPTL4 was overexpressed in OC (Fig. 1I).

### ANGPTL4 expression correlates with differential immune cell infiltration in OC

**Fig. 2:**
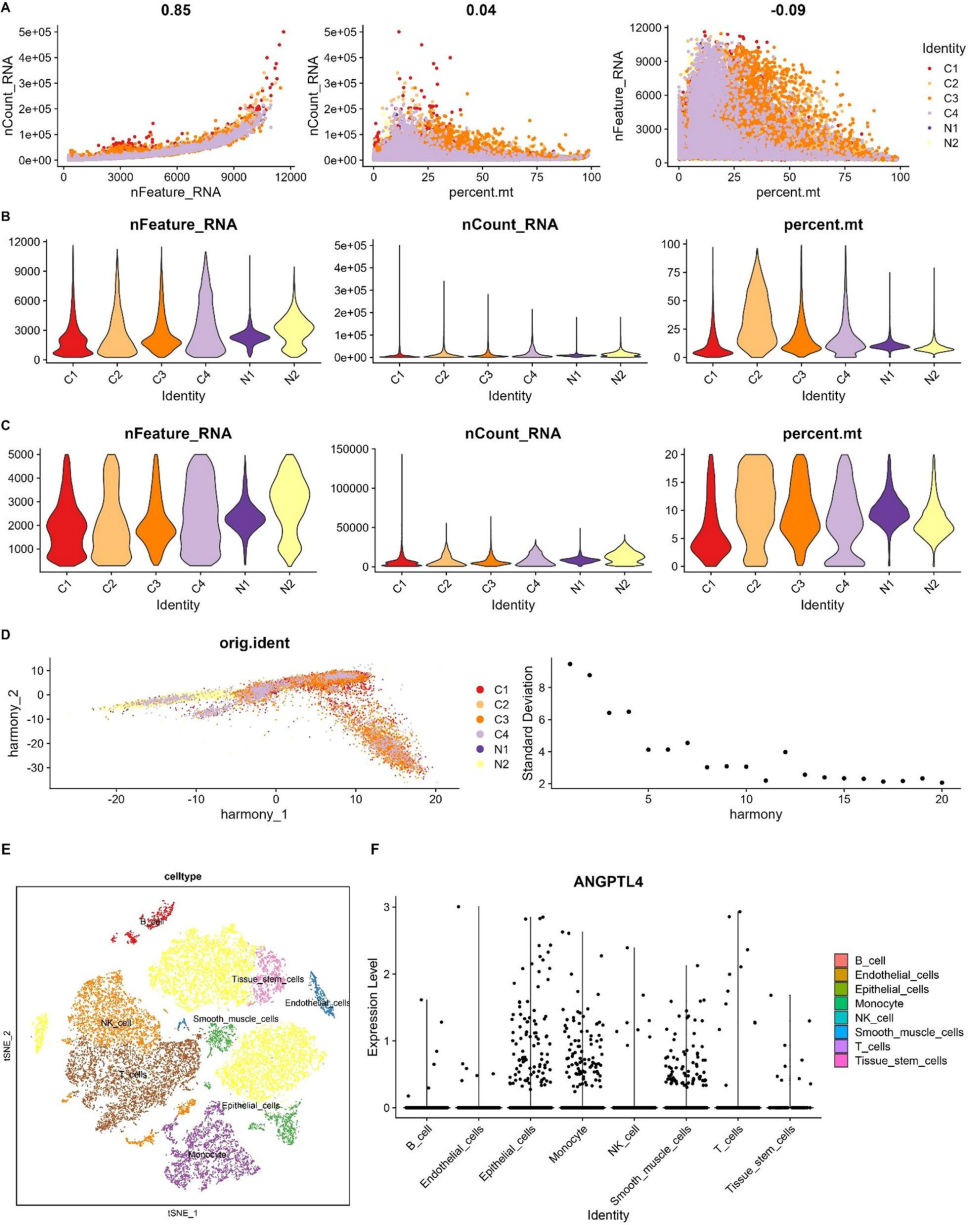
sc-RNA-seq data quality control process and annotation of cell sub-populations in GSE184880. **A** Analysis of the correlation between gene expression, cell counts, and mitochondrial content in selected samples. **B** Percent mt, nFeatureRNA, and nCountRNA for each sample before filtering. **C** nCount RNA, nFeature RNA, and percent mt for each sample after filtration. **D** The Harmony algorithm was used to integrate batch effects. Each dot in the plot represents a cell. Elbow plot visualization of principal component rankings. **E** t-Distributed Stochastic Neighbor Embedding (t-SNE) dot plot visualization of cell sub-populations. **F** Scatter plots displaying the expression of ANGPTL4 in different cell sub-populations

We next explored the potential correlation between ANGPTL4 expression and immune cell infiltration in OC. The screening process of single-cell RNA-seq profiles from OC samples in the GSE184880 dataset is depicted in Fig. 2A–E. A cell subpopulation-based analysis revealed that ANGPTL4 was highly expressed in epithelial cells and monocytes (Fig. 2F).

**Fig. 3:**
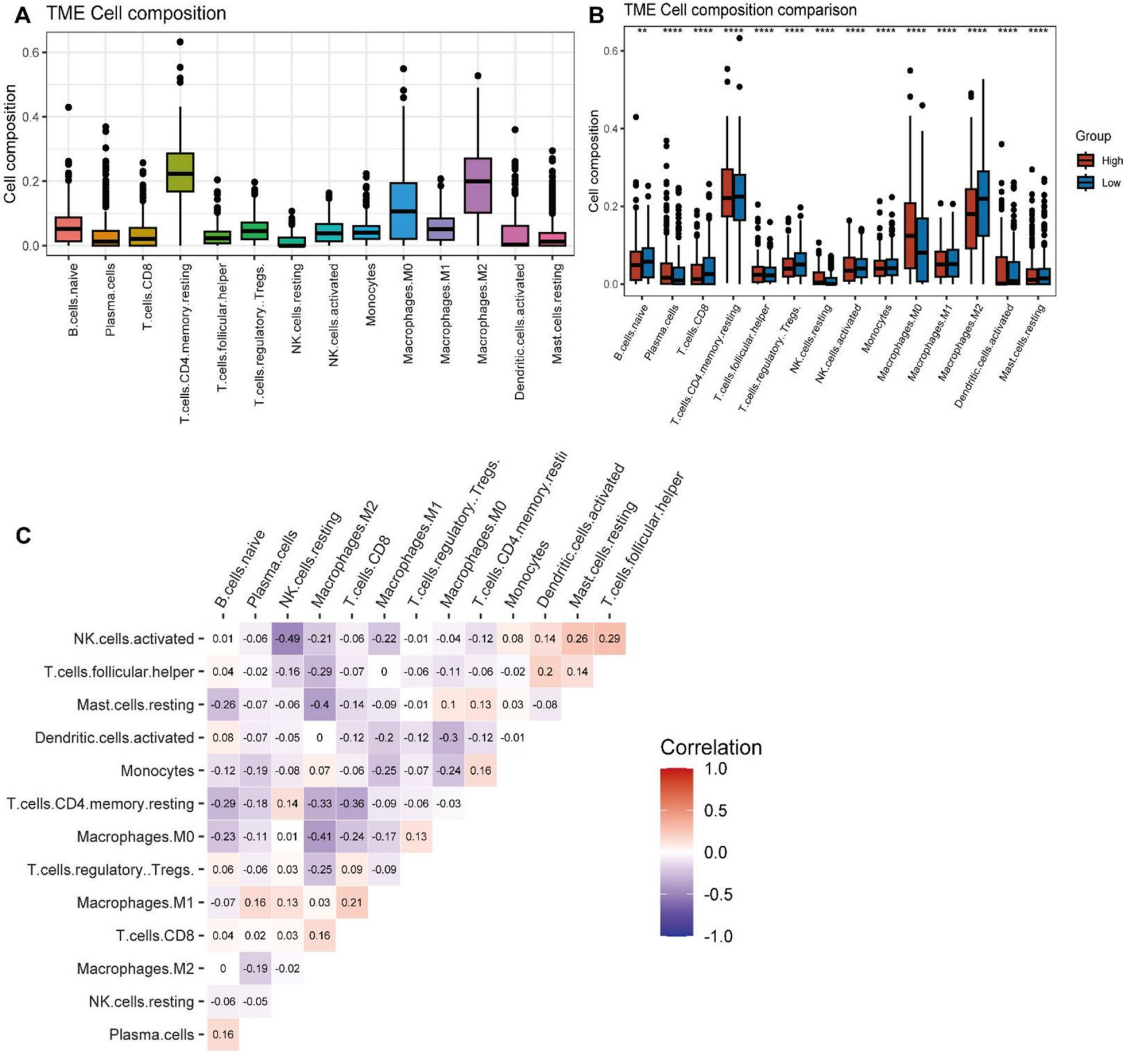
CIBERSORT analysis of OC samples in TCGA. **A** Expression of tumor-infiltrating immune cells in OC samples. **B** Proportions of infiltrating immune cells in different ANGPTL4 expression subgroups. **C** Correlation matrix of immune cell proportions

The CIBERSORT algorithm was then used to explore the potential association between ANGPTL4 expression and immune cell infiltration in OC. As shown in Fig. 3A–C, significant differences in the abundance of 14 immune cell types were detected between the low-risk and high-risk groups defined in the TCGA cohort. Specifically, the proportions of plasma cells, resting NK cells, M0 macrophages, and activated dendritic cells (aDCs) were obviously higher in the high-risk subgroup. In contrast, naive B cells, CD8 T cells, regulatory T cells, and M2 macrophages were significantly less abundant in high-risk compared to low-risk patients. Correlation analysis between immune cell subtypes indicated that activated NK cells and follicular helper T cells had the highest mutually positive relationship.

### Silencing of ANGPTL4 suppresses OC cell proliferation and migration in vitro

**Fig. 4:**
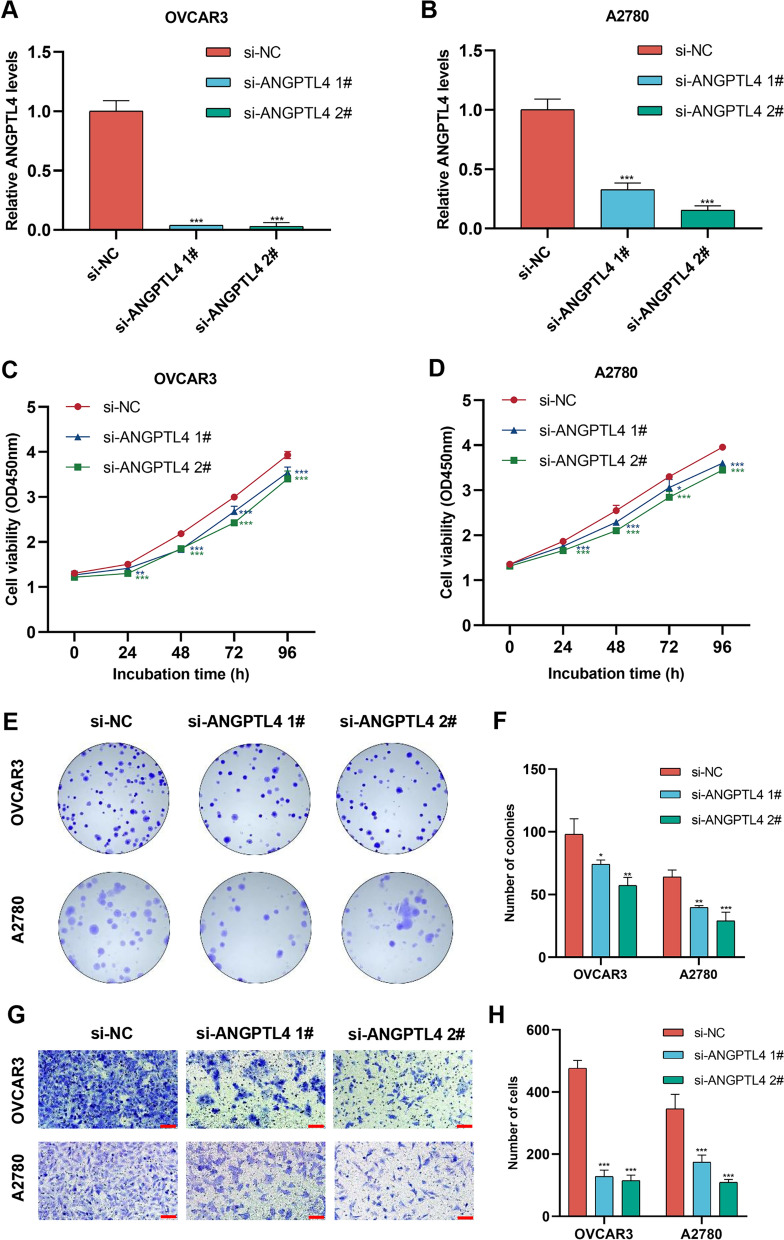
ANGPTL4 silencing suppresses the proliferative and migratory capacity of OC cells in vitro. **A**, **B** Confirmatory RT-qPCR analysis of siRNA-mediated ANGPTL4 knockdown efficiency in OVCAR3 and A2780 cells. **C**, **D**. Analysis of cell viability (CCK8 assay) in OC cells transfected with si-NC and si-ANGPTL4. **E**, **F**. Results of colony formation assays performed in OC cells transfected with si-NC and si-ANGPTL4. **G**, **H**. Transwell assays were used to detect the effect of ANGPTL4 silencing in OC cells. Scale bar = 100 μm. *p < 0.05, **p < 0.01, ***p < 0.001, compared with si-NC

To analyze the functional impact of ANGPTL4 in OC, siRNA-mediated ANGPTL4 knockdown was performed in two human OC cell lines, OVCAR3 and A2780. RT-qPCR analysis confirmed that compared to cells transfected with negative control siRNA (si-NC), the expression of ANGPTL4 was significantly downregulated in those transfected with ANGPTL4-targeted siRNA (si-ANGPTL4) (Fig. 4A, B). Subsequent experiments using CCK-8 and Transwell assays showed that silencing ANGPTL4 significantly downregulated cell proliferation and migration ability in both cell lines (Fig. 4C–H).

### Repression of ANGPTL4 inhibits OC cell proliferation in vivo

**Fig. 5:**
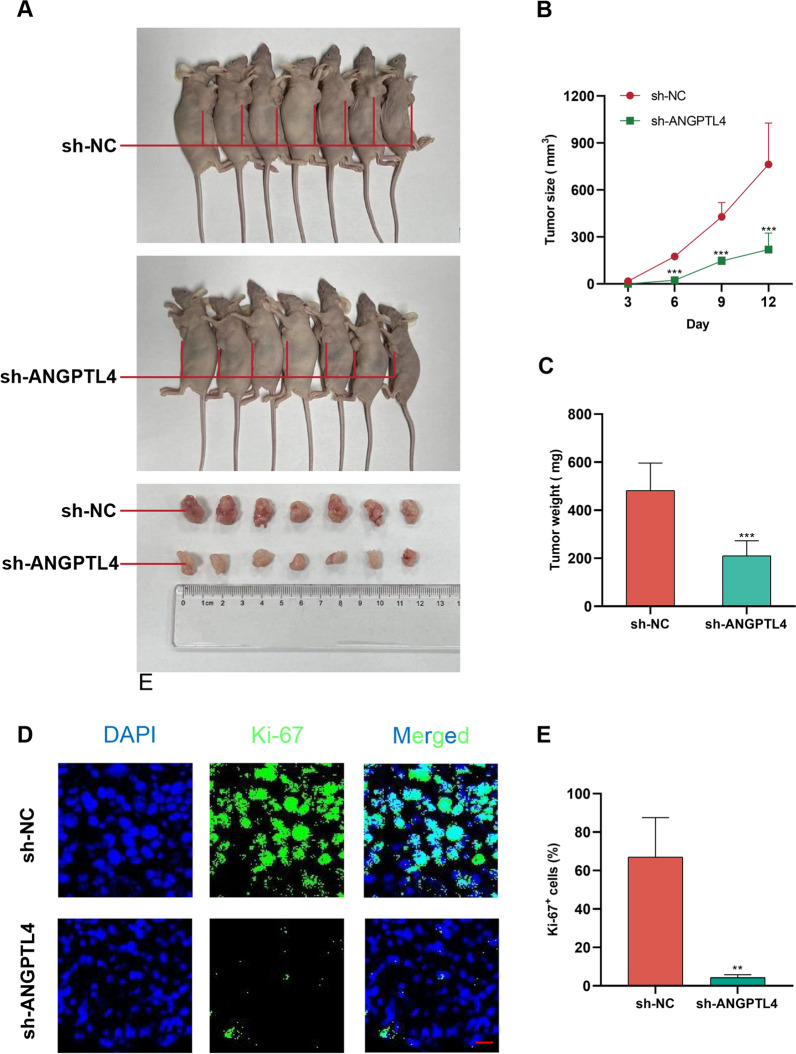
Repression of ANGPTL4 inhibits OC cell growth in vivo. **A** OVCAR3 cells transfected with sh-ANGPTL4 or sh-NC were subcutaneously injected into, respectively, the left and right armpits of nude mice. Sample size = 7. **B** Tumor growth curve. **C** Tumor weight. **D**, **E** Immunofluorescence was used to compare tumor Ki-67 expression between the sh-ANGPTL4 and sh-NC groups. Sample size = 3. Scale bar: 20 μm. **p < 0.01, ***p < 0.001 compared with sh-NC

Tumorigenesis experiments were next performed in nude mice subcutaneously injected with OVCAR3 cells transfected with sh-ANGPTL4. Control xenografts were simultaneously produced via injection of sh-NC-transfected cells in the contralateral axillae. Weight and volume measurements of tumors excised 12 days after implantation showed that those in which ANGPTL4 was silenced grew at a slower rate compared to control xenografts (Fig. 5A–C). Further suggesting a pro-tumorigenic function for ANGPTL4 in OC, immunofluorescence staining of Ki67, a widely used marker of cancer cell proliferation [30], indicated that ANGPTL4-deficient tumors exhibited much less Ki-67-positive cells than those in the sh-NC group (Fig. 5D, E).

### ANGPTL4 silencing inhibits ERK1/2 signaling in OC

**Fig. 6:**
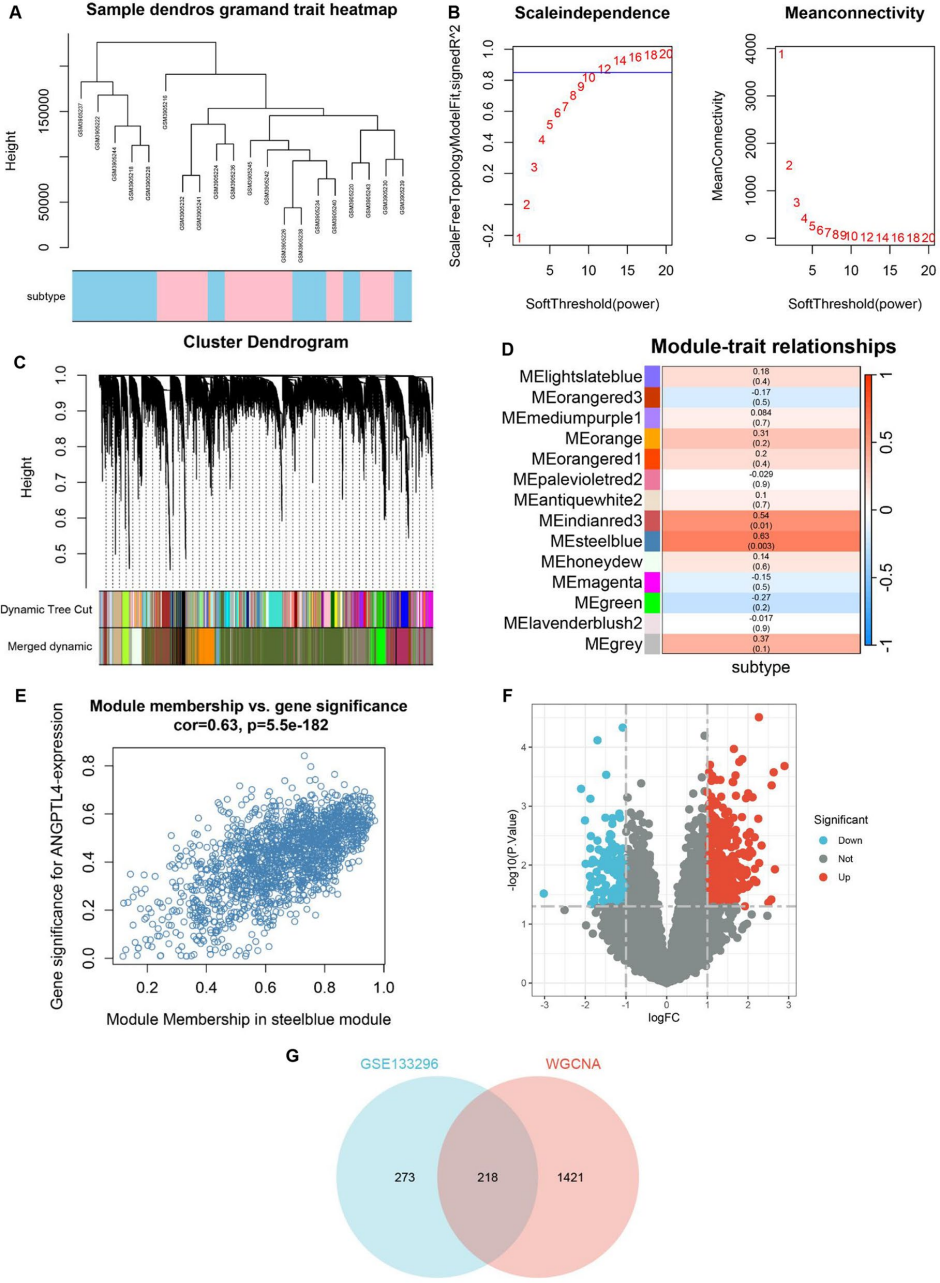
WGCNA-based detection of genes associated with ANGPTL4 expression in metastatic OC samples in GSE133296. **A** Metastasis samples in GSE133296 corresponded to the expression of ANGPTL4. **B** Calculation and selection of optimal soft-thresholding power. Influence of different powers on scale independence (left) and mean connectivity (right). **C** Module division and module merging. **D** Relationships between module traits and clinical traits. Each cell contains the corresponding correlation coefficient and p-value. **E** Scatter plots of gene significance vs. module membership in the steel blue module of the WGCNA network. **F** Volcano plot showing DEGs between the high and low ANGPTL4 expression subgroups in GSE133296. **G** Venn plot depicting the intersection between DEGs in GSE133296 and genes in the steel blue module

**Fig. 7:**
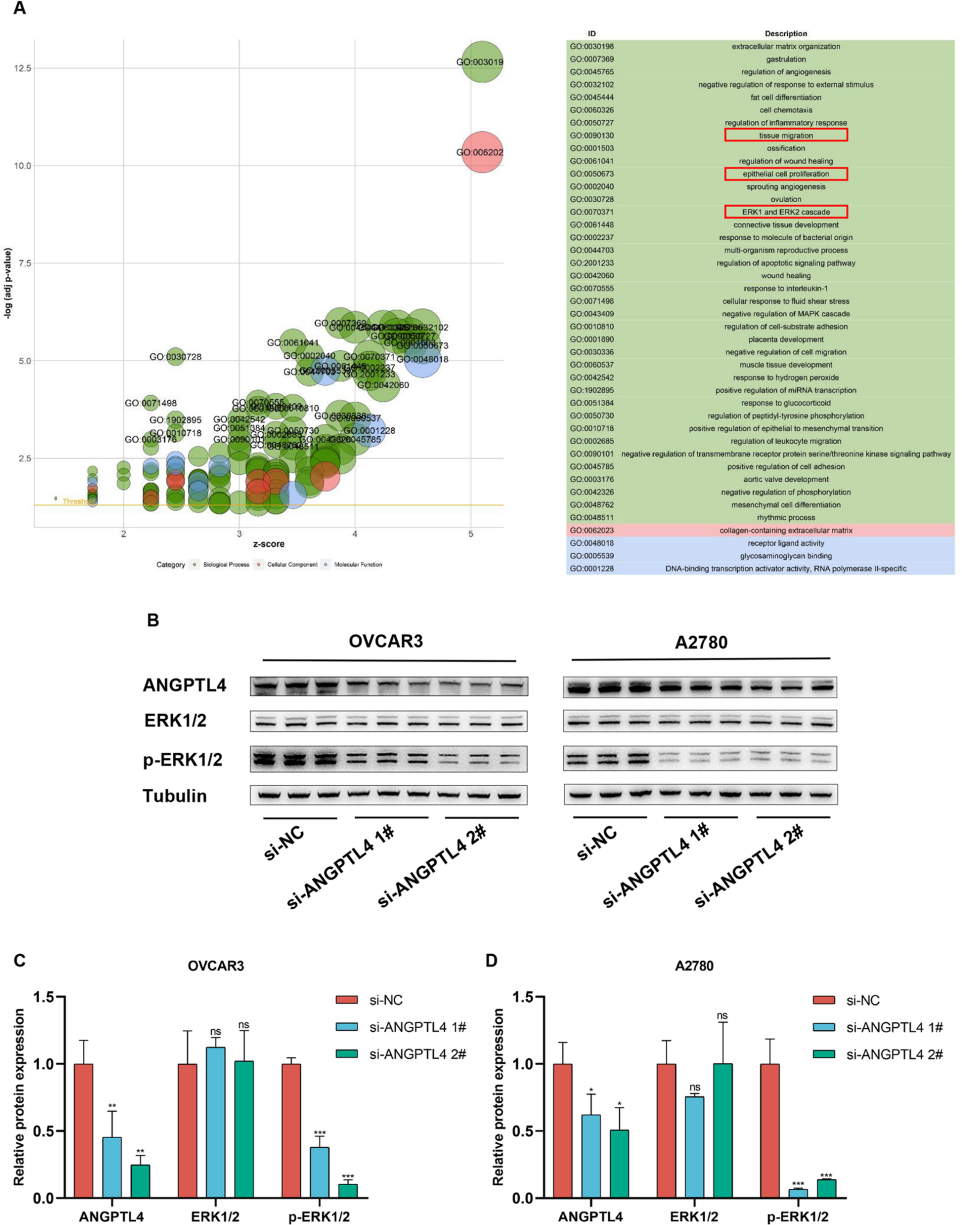
Analysis of ANGPTL4 signaling mediators in OC. **A** Results of GO enrichment analysis of intersecting genes from GSE133296 and the steel blue module. **B**–**D** Western blot analysis of ANGPTL4, p-ERK1/2, and ERK1/2 expression in two OC cells. *p < 0.05, **p < 0.01, ***p < 0.001, compared with si-NC

To evaluate potential molecular mediators of the pro-tumorigenic action of ANGPTL4, genes associated with its expression were screened by WGCNA (Fig. 6A–E). For differential expression analysis, samples in GSE133296 were then divided into two subgroups, i.e. high- and low-expression, using the median ANGPTL4 expression value as the criterion (Fig. 6F). The extracted DEGs were cross-examined against genes in the most relevant modules in WGCNA, and GO enrichment analysis was performed on the overlapping genes (n = 218) (Fig. 6G). The analysis suggested that activation of the ERK1/2 pathway by ANGPTL4 may play an important role in OC progression (Fig. 7A). ANGPTL4 regulates NSCLC progression through the ERK pathway [16]. Downregulated ANGPTL4 activates the ERK pathway thereby promoting colorectal cancer metastasis [31].

Therefore, we performed western blot analysis in cultured OVCAR3 and A2780 cells to verify ERK1/2 activity. As shown in Fig. 7B–D, the expression of total ERK1/2 remained unchanged, while that of phosphorylated ERK1/2 (p-ERK1/2) was decreased, in ANGPTL4-silenced cells. We thus speculated that ANGPTL4 knockdown may suppress OC progression through downregulation of the ERK1/2 pathway.

### ERK inhibition abrogates the progression of tumor cells

**Fig. 8:**
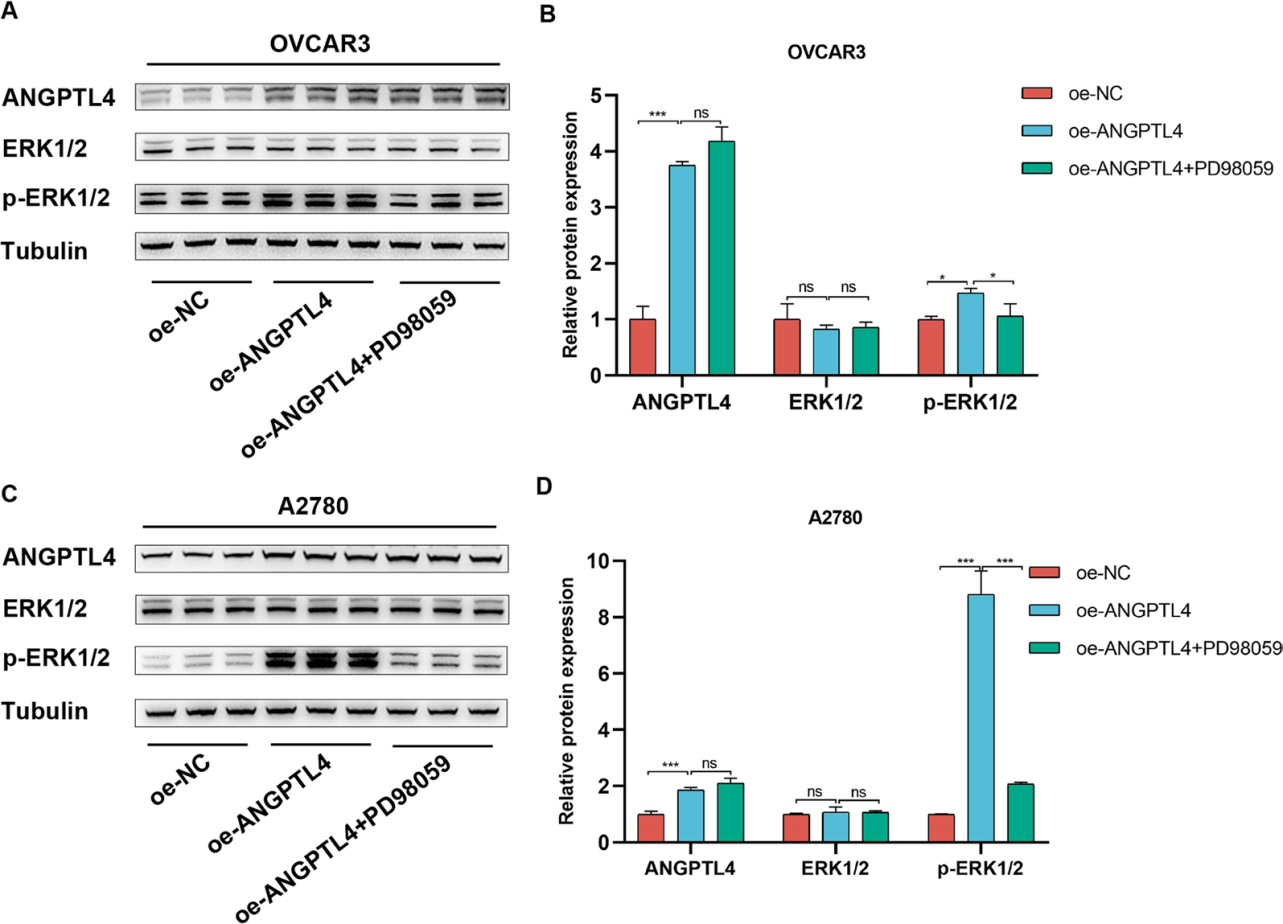
ANGPTL4 activates the ERK1/2 pathway in OC cells. **A**, **C** Western blot analysis of ANGPTL4, p-ERK1/2, and ERK1/2 expression in cells transfected with a control plasmid (oe-NC) or an ANGPTL4 overexpression plasmid (oe-ANGPTL4). PD98059 was used to inhibit ERK1/2 signaling. **B**, **D** Quantification of results from (A). *p < 0.05, **p < 0.01, ***p < 0.001, compared with oe-ANGPTL4

**Fig. 9:**
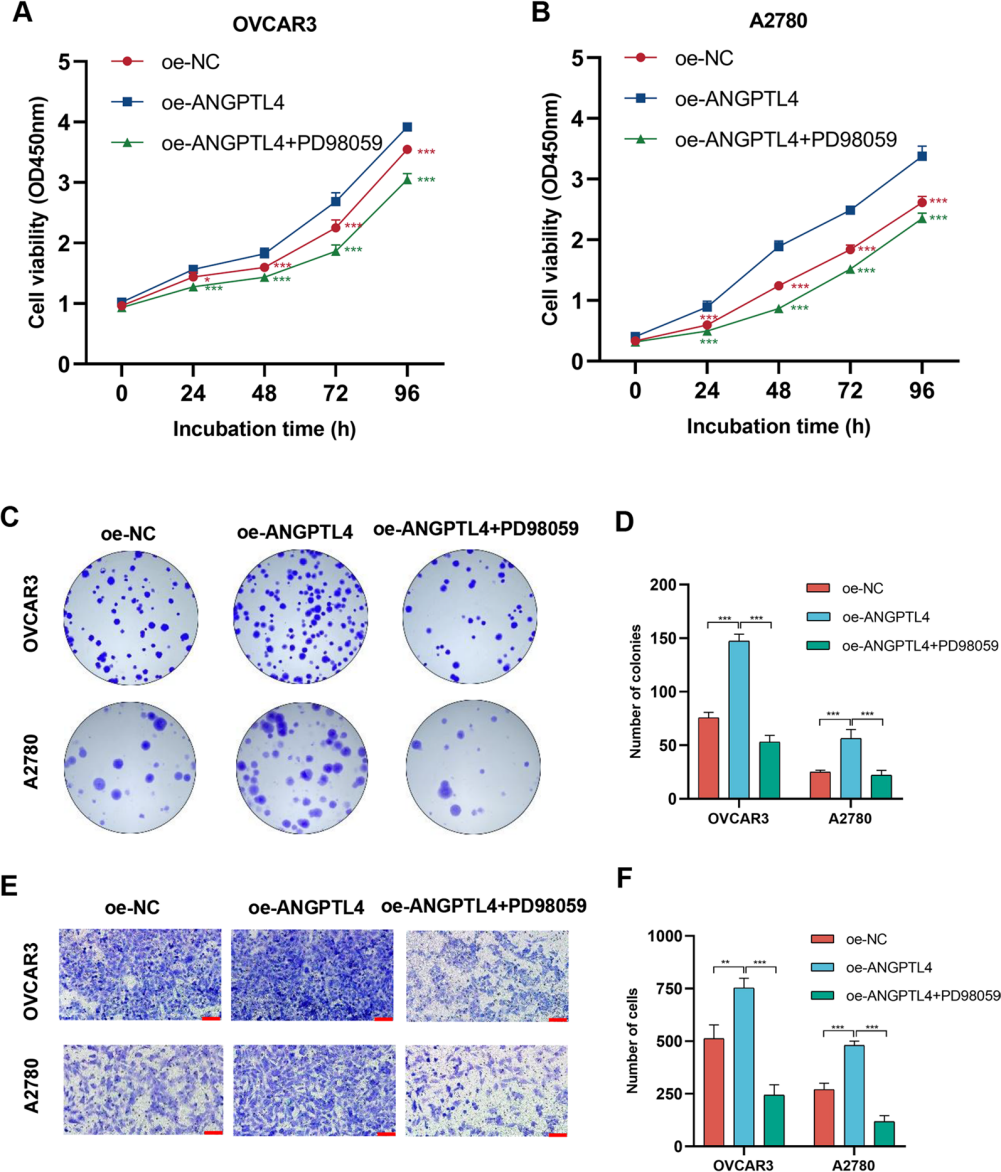
ERK inhibition abrogates growth and migration of ANGPTL4-overexpressing OC cells. **A**, **B**. CCK-8 cell proliferation assays were performed on OVCAR3 and A2780 cells in the oe-NC group, oe-ANGPTL4 group, and oe-ANGPTL4 + PD98059 group. **C**, **D**. Colony formation assay results of OVCAR3 and A2780 cells in oe-NC group, oe-AGPTL4 group, and oe-AGPTL4 + PD98059 group. **E, F.** The results of the Transwell migration assay were obtained for OVCAR3 and A2780 cells in the oe-NC group, oe-AGPTL4 group, and oe-AGPTL4 + PD98059 group.. Scale bar = 100 μm. *p < 0.05, **p < 0.01, ***p < 0.001, compared with oe-ANGPTL4

To determine whether ANGPTL4 promotes OC cell progression by modulating the ERK1/2 signaling pathway, we used PD98059, a selective inhibitor of ERK1/2 signaling, to treat cells transfected with ANGPTL4 overexpression plasmids (oe-ANGPTL4). Compared with the negative control group, the expression of p-ERK1/2 was obviously increased in the ANGPTL4 overexpression group (Fig. 8A–C). Compared to the latter group, p-ERK1/2 the expression was markedly reduced in the oe-ANGPTL4 + PD98059 group (Fig. 8A–C). Furthermore, CCK-8, colony formation, and Transwell assays showed that PD98059 exposure greatly reversed the enhancing effect of ANGPTL4 overexpression on the proliferative and migratory capacities of OC cells (Fig. 9A–F). These results suggested that ANGPTL4 regulates the malignant behavior of OC cells by stimulating the ERK1/2 signaling axis.

### ERK1/2 pathway activation leads to c-Myc, CyclinD1, and MMP2 expression in OC cells

**Fig. 10:**
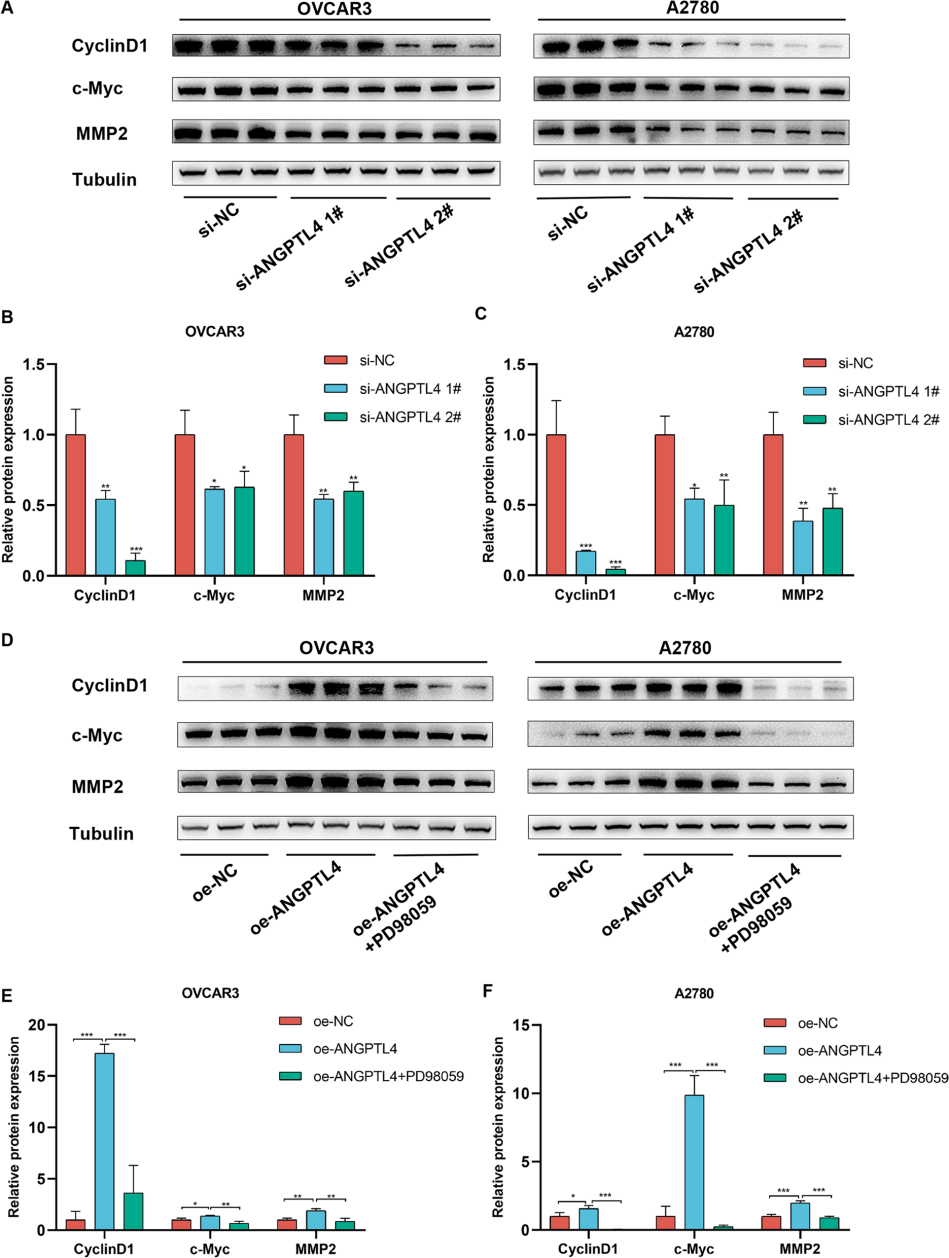
ANGPTL4 upregulates c-Myc, Cyclin D1, and MMP2 expression via ERK1/2 pathway activation. **A**–**C** Western blot analysis of c-Myc, Cyclin D1, and MMP2 expression in OC cells transfected with si-ANGPTL4. *p < 0.05, **p < 0.01, ***p < 0.001, compared with si-NC. **D**–**F** Western blot analysis of c-Myc, Cyclin D1, and MMP2 expression in OC cells overexpressing ANGPTL4. PD98059 was used to inhibit ERK1/2 signaling. *p < 0.05, **p < 0.01, ***p < 0.001, compared with oe-ANGPTL4

Results of GO enrichment analysis (Fig. 7A) indicated that ANGPTL4 was associated with tissue migration and epithelial cell proliferation. It has been shown that ERK1/2 positively regulates both proliferation-related (e.g. c-Myc, Cyclin D1) and migration-related (e.g. matrix metalloproteinase-2; MMP2) proteins to drive cancer cell progression [32–34]. Western blot results showed that when ANGPTL4 was downregulated, the levels of c-Myc, Cyclin D1 and MMP2 were reduced (Fig. 10A–C). Conversely, these proteins were all upregulated when ANGPTL4 was overexpressed, and this effect was attenuated in the presence of the ERK1/2 pathway inhibitor PD98059 (Fig. 10D–F). These results suggested that the ANGPTL4/ERK axis contributes to OC progression by positively regulating c-Myc, CyclinD1, and MMP2 expression via ERK1/2 signaling.

## Discussion

OC is a serious threat to women's lives. Hypoxia promotes tumor angiogenesis, inhibits apoptosis, and stimulates proliferation, invasion, and distant metastasis of tumor cells [35–38]; it drives also resistance to radiotherapy and surgery, thus aggravating the prognosis of OC patients [39]. Molecular targeted therapies are playing an increasingly important role in the treatment of malignant tumors. Accordingly, in recent years research has focused on novel targeted therapies to reduce OC-related mortality [40–42].

After conducting bioinformatics analysis of hypoxia-related genes in clinical OC specimens, we identified ANGPTL4 as a potential target gene. The majority of OC tissues exhibited high expression levels of ANGPTL4, as evidenced by our clinical specimens. ANGPTL4 overexpression has been detected in many human tumor cells; it is mainly secreted by proliferating tumor epithelial cells, and stimulates the downstream ERK pathway through interaction with specific integrins and extracellular matrix proteins [17, 43, 44]. Hypoxia-driven ANGPTL4 expression facilitates tumor growth and metastasis [45], and may thus serve as both a molecular marker for the diagnosis of OC and a target for drug discovery. In this work, the oncogenic role of ANGPTL4 was demonstrated through in vitro and in vivo assays which showed that ANGPTL4 deficiency suppressed OC cell proliferation and migration and inhibited the growth of OC xenografts in nude mice. Based on these findings, we concluded that ANGPTL4 may represent a novel target for the diagnosis and treatment of OC.

Under hypoxic conditions, increased expression of genes involved in tumor progression leads to radiotherapy and drug resistance and immune tolerance [46–49]. We explored the correlation between ANGPTL4 expression and immune cell infiltration in OC, and found that ANGPTL4 was highly expressed in monocytes and epithelial cells. Interestingly, the CIBERSORT algorithm indicated differential representation of 14 tumor-infiltrating immune cell types between OC cases segregated into low- and high-risk groups. In particular, the abundance of plasma cells, resting NK cells, M0 macrophages, and aDCs was significantly higher in the high-risk group. DCs recognize and process tumor antigens and activate tumor-specific T-cell responses, which are key determinants of tumor immunity [50, 51]. Under pathological conditions, various suppressive cytokines present in the tumor microenvironment act on DCs, impairing their function and thus contributing to the escape of tumor cells from immune surveillance [52, 53]. It has been found that advanced ovarian tumors in humans and mice are heavily infiltrated by DCs that lack antigen-presenting capacity and exhibit immunosuppressive activity, which prevents local activation and expansion of T cells [54, 55]. Consistent with our findings, Yang and colleagues found significant overabundance of M0 macrophages in a high-risk, poor-prognosis OC patient population [56]. In contrast, the previously reported association between tumor-infiltrating plasma cells and good prognosis in OC is in apparent conflict with the present results [57], requiring therefore further exploration.

Through both bioinformatics analysis and in vitro experiments, the mechanisms by which ANGPTL4 regulates OC cell progression were investigated in this work. Results of WGCNA, subgroup expression, and GO enrichment analyses suggested that activation of the ERK1/2 pathway by ANGPTL4 plays an important role in OC progression. ERK1/2 activation mediates phosphorylation of phosphatases, cytoskeletal proteins, and transcriptional factors to regulate cell differentiation, proliferation, and migration [58]. Overactivation of ERK1/2 signaling enhances cell proliferation and promotes malignant transformation [34]. Indeed, research has shown that activation of the ERK1/2 pathway contributes to ovarian tumor growth [32, 59, 60]. In our study, the expression of phosphorylated ERK1/2 was significantly reduced after ANGPTL4 knockdown or ERK1/2 inhibition with PD98059. In turn, proliferation and migration were impaired when PD98059 was applied to ANGPTL4-overexpressing cells. Therefore, we inferred that ANGPTL4 regulates OC cell growth and motility by activating the ERK1/2 pathway.

**Fig. 11:**
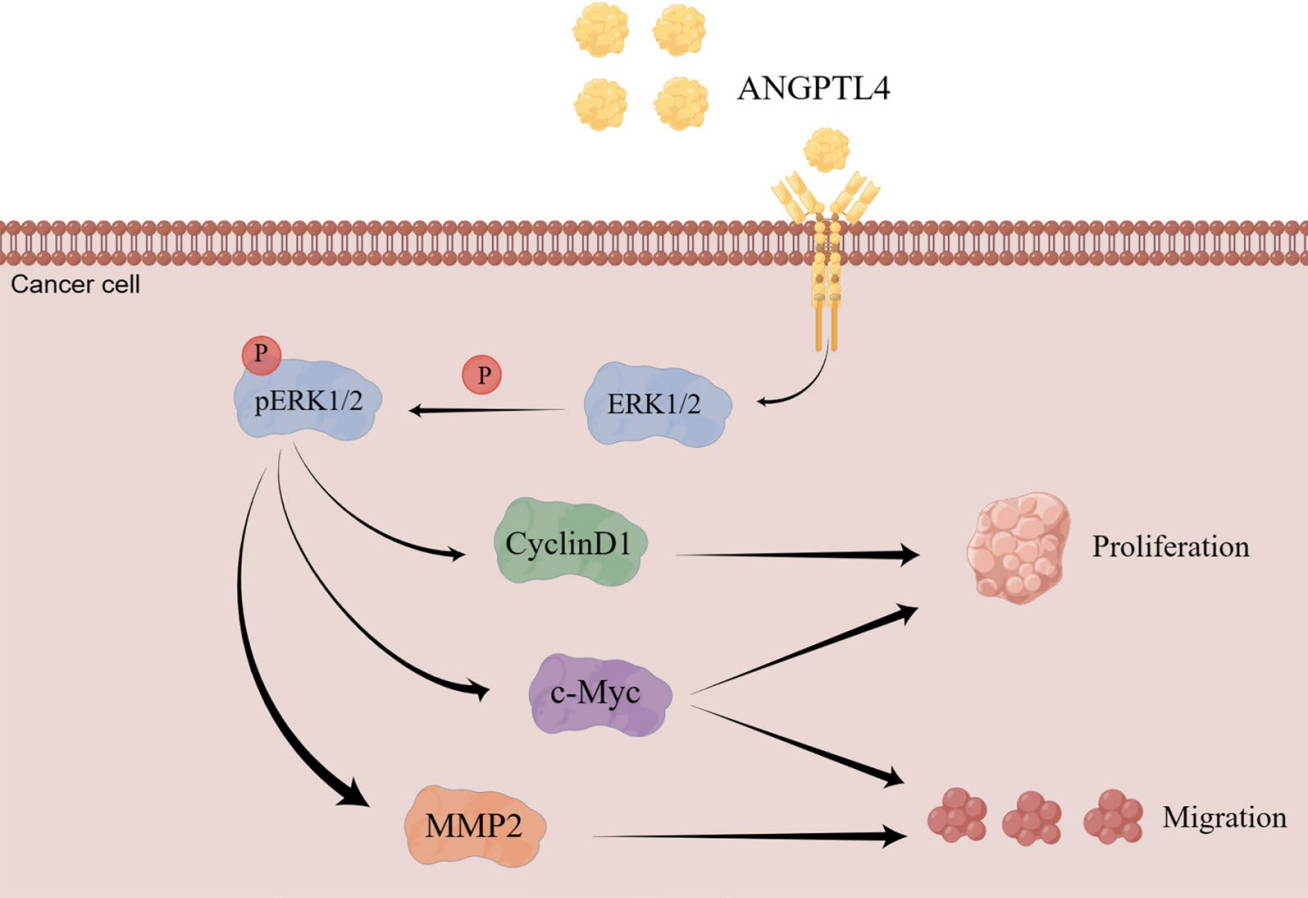
Schematic depiction of potential mechanisms by which ANGPTL4-ERK1/2 signaling regulates OC progression. (Drawing by Figdraw; www.figdraw.com)

We further investigated the potential mediators that act downstream of ANGPTL4/ERK to regulate the proliferation and migration of OC cells. We found that in ANGPTL4-deficient cells decreased expression of p-ERK1/2 was accompanied by reduced expression of CyclinD1, c-Myc, and MMP2. As downstream targets of ERK1/2 signaling, c-Myc and CyclinD1 are crucial regulators of cell proliferation [61]. The proto-oncogene c-Myc encodes a transcription factor that critically regulates cell division, proliferation, differentiation, migration, and programmed cell death [62–64]. Cyclin D1, a highly conserved member of that cyclin protein family, regulates cell cycle at the G1/S transition phase [65] and its expression has been reported to stimulate proliferation of OC cells [66, 67]. The ERK/MMP2 pathway is one of the key triggers of tumor cell migration in human cancers [32, 68, 69]. Studies have shown that MMPs influence tumor cell migration, invasion and metastasis, particularly in advanced ovarian serous cancers [70, 71], and a correlation between MMP2 expression and OC progression has been established [72, 73]. In this study, ANGPTL4 knockdown and overexpression experiments, along with selective ERK1/2 signaling inhibition via PD98059, confirmed that the ANGPTL4/ERK pathway regulates the expression of CyclinD1, c-Myc, and MMP2 in OC cells. Based on these findings, we speculated that ANGPTL4/ERK signaling promotes OC cell proliferation and migration by upregulating CyclinD1, c-Myc, and MMP-2 expression (Fig. 11).

One limitation of this study is the absence of a specific mechanism elucidating the regulation of c-Myc, CyclinD1, and MMP2 by ANGPTL4-ERK1/2 in OC, which will be further investigated in our future study. Additionally, the study is limited by the use of clinical specimens from a single source, which were relatively small in number. Thus, a further study with a larger sample size is warranted to address this limitation. Nevertheless, this study significantly contributes to our comprehension of the role of ANGPTL4 in OC, as it is the first to elucidate the involvement of ANGPTL4-ERK1/2 signaling in the progression of OC (Additional file 2).

## Conclusion

In summary, our in vitro and in vivo experiments suggest that upregulation of the hypoxia-associated ANGPTL4 gene promotes OC proliferation and migration. Mechanistically, ANGPTL4 activates the ERK1/2 signaling pathway, leading to the upregulation of c-Myc, CyclinD1, and MMP2 expression, thereby facilitating the progression of OC. This investigation into the mechanisms underlying the ANGPTL4-ERK1/2-c-Myc/CyclinD1/MMP2 pathway holds promise for improving OC diagnosis and treatment strategies.

### Supplementary Information

**Additional file 1: Table S1.** Antibodies information.

**Additional file 2:** Raw Data

## Data Availability

The datasets analyzed in the current study are publicly available from their respective sources. The data that support the findings of this study are available from the corresponding author upon reasonable request.
